# Novel Sources of Stripe Rust Resistance Identified by Genome-Wide Association Mapping in Ethiopian Durum Wheat (*Triticum turgidum* ssp. *durum*)

**DOI:** 10.3389/fpls.2017.00774

**Published:** 2017-05-12

**Authors:** Weizhen Liu, Marco Maccaferri, Sheri Rynearson, Tesfaye Letta, Habtemariam Zegeye, Roberto Tuberosa, Xianming Chen, Michael Pumphrey

**Affiliations:** ^1^Department of Crop and Soil Sciences, Washington State University, PullmanWA, USA; ^2^Department of Agricultural Sciences, University of BolognaBologna, Italy; ^3^Sinana Agricultural Research CenterBale-Robe, Ethiopia; ^4^Ethiopian Institute of Agricultural ResearchAddis Ababa, Ethiopia; ^5^Wheat Health, Genetics, and Quality Research Unit, Agricultural Research Service (USDA), PullmanWA, USA; ^6^Department of Plant Pathology, Washington State University, PullmanWA, USA

**Keywords:** wheat landraces, Ethiopian tetraploid wheat, association analysis, yellow rust resistance, 90 K wheat SNP array

## Abstract

Stripe rust of wheat, caused by *Puccinia striiformis* f. sp. *tritici* (*Pst*), is a global concern for wheat production, and has been increasingly destructive in Ethiopia, as well as in the United States and in many other countries. As Ethiopia has a long history of stripe rust epidemics, its native wheat germplasm harbors potentially valuable resistance loci. Moreover, the Ethiopian germplasm has been historically underutilized in breeding of modern wheat worldwide and thus the resistance alleles from the Ethiopian germplasm represent potentially novel sources. The objective of this study was to identify loci conferring resistance to predominant *Pst* races in Ethiopia and the United States. Using a high-density 90 K wheat single nucleotide polymorphism array, a genome-wide association analysis (GWAS) was conducted on 182 durum wheat landrace accessions and contemporary varieties originating from Ethiopia. Landraces were detected to be more resistant at the seedling stage while cultivars were more resistant at the adult-plant stages. GWAS identified 68 loci associated with seedling resistance to one or more races. Six loci on chromosome arms 1AS, 1BS, 3AS, 4BL, and 5BL were associated with resistance against at least two races at the seedling stage, and five loci were previously undocumented. GWAS analysis of field resistance reactions identified 12 loci associated with resistance on chromosomes 1A, 1B, 2BS, 3BL, 4AL, 4B and 5AL, which were detected in at least two of six field screening nurseries at the adult-plant stage. Comparison with previously mapped resistance loci indicates that six of the 12 resistance loci are newly documented. This study reports effective sources of resistance to contemporary races in Ethiopia and the United States and reveals that Ethiopian durum wheat landraces are abundant in novel *Pst* resistance loci that may be transferred into adapted cultivars to provide resistance against *Pst*.

## Introduction

Stripe rust (also called yellow rust), caused by the fungal pathogen *Puccinia striiformis* Westend. f. sp. *tritici* Erikss. (*Pst*), is a major foliar disease in most wheat-growing regions worldwide. Regional yield loss caused by *Pst* has regularly ranged from 0.1 to 5% and in rare events up to 25% (Wellings, 2011). However, yield loss of individual susceptible wheat cultivars can be 100% if the infection is established in the early growth stages and epidemic development continues throughout the wheat-growing season (Chen, 2005). To protect against stripe rust-associated losses, developing and growing resistant cultivars is widely recognized as the most environmentally and economically feasible approach. International wheat breeding programs at International Maize and Wheat Improvement Center (CIMMYT) and International Center for Agricultural Research in the Dry Areas (ICARDA) have released high yielding, widely adapted germplasm with rust resistance and distributed these elite cultivars globally (Rajaram et al., 1992; Byerlee and Moya, 1993; Rajaram, 2001). However, the resistance of many of these cultivars was based on single race-specific all-stage resistance genes, which confer a high level of resistance to specific races throughout all growth stages. When used extensively over time and space, single resistance genes generally loose their effectiveness in a few years and the cultivars are vulnerable to stripe rust epidemics (Line and Chen, 1995; Chen, 2005, 2007). A recent case was observed in 2009 and 2010, when *Pst* pathotypes overcame the resistance conferred by *Yr27* in a number of Central and West Asian and Northern African countries, resulting in a more than US$3.2 million for fungicides in Ethiopia and high yield loss in Syria, Morocco, Iran, and Turkey (Solh et al., 2012; Zegeye et al., 2014). Moreover, in a countrywide *Pst* virulence survey in Ethiopia during 2013–2014, Wan et al. (2016) identified 18 *Pst* races from bread and durum wheat, and 14 of them represented virulence combinations not previously reported. All Ethiopian races detected were virulent to resistance genes *Yr8* and *Yr9*, which is characteristic of increased-aggressiveness races of post-2000 populations now found throughout the world (Milus et al., 2009). These new and more aggressive races pose severe threats to the wheat production of Ethiopia and the neighboring countries in the East Africa and have motivated wheat breeders to search for additional resistance sources for long-term control.

Deployment of race-nonspecific resistance, also called slow rusting, adult plant, or partial resistance, is another option to improve stripe rust resistance of wheat varieties. Race-nonspecific resistance is often controlled by multiple additive loci of minor effects and is inherited quantitatively, but single genes for race-nonspecific resistance have also been found, for example, *Yr36* (Fu et al., 2009) and *Lr34*/*Yr18* (Krattinger et al., 2009). Race-nonspecific resistance is believed to be more durable than race-specific resistance. A high level of resistance or approaching immunity can be achieved in plants at later growth stages when pyramiding four to five such genes (Singh et al., 2000, 2005). The development of molecular markers tightly linked with adult plant resistance (APR) genes will enable gene pyramids that can be routinely used in marker-assisted selection (MAS) breeding. However, to date, only a few genes including *Lr34*/*Yr18* (Lagudah et al., 2006), *Lr67*/*Yr46* (Herrera-Foessel et al., 2014) and *Yr36* (Distelfeld et al., 2006) have reliable diagnostic markers.

Durum wheat (2n = 4x = 28, AABB) is the second most widely cultivated wheat crop, which currently accounts for 8% of global wheat production (Mengistu and Pè, 2016). Breeding activities are concentrated in the Mediterranean region, Eastern Europe and Northern America (Royo et al., 2009). However, traditional farmer varieties, commonly referred as landraces, are preserved in several agro-ecosystems of the Old World, especially in Ethiopia which is considered a center of tetraploid wheat diversity (Vavilov, 1951; Harlan, 1969; Vavilov and Dorofeev, 1992; Badebo et al., 2009). In Ethiopia, durum wheat is long established, as it was likely introduced into the northern highlands of Ethiopia around 3,000 BC (Badebo et al., 2009). There, durum is cultivated in traditional small-to-medium scale farming systems in a diverse geographical (altitude varying from 1,600 to 3,000 m) and climatic conditions (Alamerew et al., 2004) and seeds are exchanged between families and villages informally. An estimated 85% of durum wheat cultivars grown in Ethiopia in recent decades are landraces (Bechere et al., 1996; Badebo et al., 2009) and over 7,000 accessions have been conserved in the Ethiopian Biodiversity Institute gene bank (Mengistu and Pè, 2016). The investigation of genetic diversity has been initiated in Ethiopian durum wheat landraces at both morphological and molecular levels (Alamerew et al., 2004; Mengistu et al., 2016), and confirmed the uniqueness of Ethiopian wheat to various degrees (Alamerew et al., 2004; Gashaw et al., 2007; Hailu et al., 2010; Mengistu et al., 2016). Although, Porceddu et al. (1975) reported Ethiopian landraces were unique sources for rust resistance in early years, no recent studies have characterized the genetic architecture of rust resistance using the genome-wide high-density genetic markers.

The recent availability of genotyping-by-sequencing (GBS) (Davey et al., 2011; Poland et al., 2012) and high-density single nucleotide polymorphism (SNP) wheat chips including Illumina^®^ iSelect 9 and 90 K SNP assays (Cavanagh et al., 2013; Wang et al., 2014) provides sufficient molecular markers for genome-wide association mapping (GWAS) that has a higher mapping resolution and higher potential to identify diagnostic markers than the traditional linkage mapping methods (Zhu et al., 2008; Brachi et al., 2011). The aims of this study were: (1) to access the diversity of stripe rust resistance in a collection of Ethiopian durum wheat, (2) to understand the genetic architecture of resistance loci at the genome-wide level, (3) to detect novel seedling and field resistance loci conferring resistance against a contemporary spectrum of *Pst* races that could be exploited to improve the resistance level of elite durum and bread wheat, and (4) to identify SNP markers significantly associated with these resistance loci to facilitate MAS.

## Materials and Methods

### Plant Materials

A total of 197 spring durum wheat accessions that are maintained by the Debre Zeit Agricultural Research Center (DZARC) and Sinana Agricultural Research Center (SARC) as single seed descent (SSD) progenies were used to assemble the association mapping panel. The panel was genotyped using the 90 K Illumina iSelect wheat SNP array (Wang et al., 2014) and 182 accessions with missing marker data frequencies less than 10% were used in GWAS analyses (Supplemental Data Sheet 1). These accessions are cultivated in different wheat-producing regions of Ethiopia, including Wello, Akaki, Bichena, Gojam, Gonder, Tigray, Shoa and Bale, as well as 11 lines that are now cultivated in the United States. The collection includes 160 landraces and 22 cultivars released by DZARC or SARC.

### Phenotypic Data

Four *Pst* races (PSTv-14, PSTv-37, PSTv-40, and PSTv-51) (Wan and Chen, 2014) collected from bread wheat in the United States and two races (PSTv-106 and PSTv-110) collected from bread and durum wheat in Ethiopia (**Table 1**, Wan et al., 2016) were chosen to conduct single-race seedling test experiments. Three seeds for each of the 197 accessions were planted in 96-well trays containing soil mix (6 L peat moss, 2 L perlite, 3 L sand, 3 L potting soil mix, 4 L vermiculite, 100 g Osmocote, and 2 L water). ‘AvS’ was included in each tray to serve as a susceptible check. To confirm the race identity, a core set of *Pst* differentials containing 18 *Yr*-single gene lines (Wan and Chen, 2014) was included in each single-race test. Seedlings were grown under controlled greenhouse conditions (a diurnal temperature cycle of 20–24°C, a nocturnal cycle of 15–18°C and 16 h photoperiod). When the second leaves were fully expanded, the plants were inoculated with a single race and incubated in a 10°C dark dew chamber with 100% relative humidity for 24 h. Then, seedlings were transferred to a rust-free growth chamber at a diurnal temperature cycle gradually changing from 4°C at 2:00 am to 20°C at 2:00 pm and 16 h light and 8 h dark and grown for 18–20 days before recording the infection type (IT) data based on the 0–9 scale (Line and Qayoum, 1992). ITs of three seedlings for each accession were recorded as one score if reactions were the same. If different reactions were present, accessions were re-tested against the *Pst* races. Accessions with IT from 0 to 3 were considered as resistant, IT from 4 to 6 as intermediate and IT from 7 to 9 as susceptible. Resistant accessions were re-tested to verify their resistance to the corresponding *Pst* races.

**Table 1:** *Puccinia striiformis* f. sp. *tritici* (*Pst*) races used for seedling tests under controlled greenhouse conditions.

*Pst* race (isolate)^a^	Origin	Virulence/avirulence formula on *Yr* genes^b^
PSTv-14 (11-116-NG)	US	***1,6,7,8,9,17,27,43,44,Tr1, Exp2,Tye***/*5,10,15,24,32,SP*
PSTv-37 (12-114-NG)	US	***6,7,8,9,17,27,43,44,Tr1, Exp2***/*1,5,10,15,24,32,SP,Tye*
PSTv-40 (09-78)	US	***6,7,8,9,10,24,27,32,43,44, Tr1,Exp2***/*1,5,15,17,SP,Tye*
PSTv-51 (11-366)	US	***1,6,7,8,9,10,17,24,27,32,43, 44,SP,Tr1,Exp2,Tye***/*5,15*
PSTv-106 (ET13-36)	Ethiopia	***1,6,7,9,17,27,43,44,Exp2***/ *5,8,10,15,24,32,SP,Tr1,Tye*
PSTv-110 (ET13-4)	Ethiopia	***1,6,7,8,9,10,17,24,27,32, 43,44,Exp2***/*5,15,SP,Tr1,Tye*

*^a^11-116-NG,12-114-NG, 09-78, 11-366, ET13-36, and ET13-4 are the isolate numbers of PSTv-14, PSTv-37, PSTv-40, PSTv-51, PSTv-106, and PSTv-110 inoculated on the 182 Ethiopia durum wheat landrace accessions, respectively. ^b^The virulence/avirulence formula was developed according to reactions of the 18 *Yr* near isogenic lines in the ‘Avocet S’ background to *Pst* in the US: *1* = AvSYr1NIL (*Yr1*); *5* = AvSYr5NIL (*Yr5*); *6* = AvSYr6NIL (*Yr6*); *7* = AvSYr7NIL (*Yr7*); *8* = AvSYr8NIL (*Yr8*); *9* = AvSYr9NIL (*Yr9*); *10* = AvSYr10NIL (*Yr10*); *17* = AvSYr17NIL (*Yr17*), *24* = AvSYr24NIL (*Yr24*); *27* = AvSYr27NIL (*Yr27*); *32* = AvSYr32NIL (*Yr32*); *43* = AvS/IDO377s (F3-41-1) (*Yr43*); *44* = AvS/Zak (1-1-35-line 1) (*Yr44*); *SP* = AvSYrSPNIL (*YrSP*); *Tr1* = AvSYrTres1NIL (*YrTr1*); *Exp2* = AvS/Exp 1/1-1 line 74 (*YrExp2*); *Tye* = Tyee (*YrTye*) (Wan and Chen, 2014). The *Yr* genes (differentials) with bold and italic values in virulence/avirulence formula indicated virulence reactions to the corresponding *Pst* races.*

To evaluate the reactions to *Pst* challenge at adult-plant stages, the 197 accessions and checks were planted in single rows without replicates in six environments of the US Pacific Northwest under natural infection of *Pst*. The predominant races in the local environments are presented in Supplementary Table S1. These environments included Whitlow farm located in eastern Washington State (WA), USA (46.721275° N, 117.150170° W) in 2014 (WHT14); Spillman Farm located in eastern WA (47.467413° N, 122.931436° W) in 2014 (SPM14) and 2015 (SPM15); Mount Vernon located in western WA (48.421215° N, 122.334047° W) in 2014 (MTV14) and 2015 (MTV15), and Central Ferry located in central WA (46.644795° N, 117.781377° W) in 2015 (CLF15). Spring wheat variety ‘Avocet Susceptible’ (AvS), known to be susceptible to stripe rust at both the seedling and adult-plant stages, was used as susceptible check and planted every 20 rows. Another susceptible variety, ‘Lemhi,’ was planted around each trial as borders to ensure the uniformity of *Pst* inoculum. For each accession, approximately 150 seeds were planted in rows of 0.5 m long and 0.3 wide. Two disease phenotypes, IT and disease severity (SEV, rating as a percentage of diseased areas covering flag leaves with a 0–100% scale) (Line and Qayoum, 1992), were recorded when the flag leaves of susceptible check ‘AvS’ were rated with IT from 7 to 9 and SEV from 70 to 100%. Since plant height and heading date might play a role in the stripe rust resistance phenotypes during adult-plant stages, these two traits were also scored in the field during the growing seasons of 2014 and 2015.

### Phenotypic Data Analyses

Best linear unbiased predictors (BLUPs) for IT and SEV of each line across six environments were calculated using PROC MIXED statement in SAS v.9.3 (SAS Institute, Inc., Cary, NC, USA).

Analysis of variance (ANOVA) was computed using PROC MIXED COVTEST statement in SAS v.9.3 treating genotypes, environments and genotype by environment interactions as random factors. The broad-sense heritability (*H^2^*) were calculated using the formula:

$$\begin{array}{c} H^{2}{}^{\;}=\;\sigma_{G}{}^{2}/[\sigma_{G}{}^{2}\;+\;(\sigma_{E}{}^{2}\;+\;\sigma_{G}{*}_{E}{}^{2}\;+\;\;\sigma_{e}{}^{2})/n] \end{array}$$

where σ_G_^2^ = variance component of genotypes, σ_E_^2^ = variance component of environments, σ_G_∗_E_^2^ = variance of environment and genotype interactions, σ_e_^2^ = variance of residuals, n = number of environments.

Pearson’s correlation coefficients of IT and SEV among six environments were analyzed using PROC CORR statement in SAS v.9.3.

### Genotypic Data

The tissue samples of each accession were collected from 25 7-day-old seedlings that were the same source of seeds used in the phenotypic evaluations. The genomic DNA was extracted using the DNeasy 96 Plant Kit (Qiagen GmbH, Hilden, Germany). SNP markers used in this study were generated using the Illumina^®^ iSelect 90 K wheat SNP assay (Wang et al., 2014). Marker genotypes were called with the GenomeStudio v2011.1 software package (Illumina, San Diego, CA, USA). Before exporting genotype data from GenomeStudio, calls showing residual heterozygousity were entered as missing values. Individuals with >15% missing SNP calls and markers with >10% missing and <10% minor allele frequency (MAF) were eliminated. The chromosome positions of SNPs were based on a high-density consensus map of tetraploid wheat generated by Maccaferri et al. (2015a). Markers with unknown chromosome positions were removed. After filtering the genotypic data, 182 accessions and 7,867 polymorphic markers were retained for the following analyses. Among 7,867 SNPs, 3,464 and 4,403 SNPs were located in A and B genomes, respectively.

Tightly-linked or diagnostic markers for *Yr5, Yr15, Yr36*, and *Yr30/Sr2* were genotyped on the current panel, including SNP-derived Kompetitive allele-specific PCR (KASP) maker-IWA6121 and IWA4096 (Naruoka et al., 2016) for *Yr5*, barc8 for *Yr15* (Yaniv et al., 2015), uhw89 for *Yr36* (Distelfeld et al., 2006) and SNP-derived KASP marker wMAS000005^1^ for *Yr30*/*Sr2.* The primer information of these diagnostic markers is provided in Supplementary Table S2.

### Population Structure and Kinship

To analyze population structure, Bayesian model-based clustering and distance-based hierarchical clustering methods were performed in STRUCTURE v.2.3.4 (Pritchard et al., 2000; Falush et al., 2003) and JMP^®^ Genomics v6.0 (SAS Institute, Inc., Cary, NC, USA 2012), respectively. For population structure analysis, 1,086 tag-SNPs were selected by a linkage disequilibrium (LD)-based method implemented in HAPLOWVIEW v4.2 (Barrett et al., 2005) with a setting of *r^2^* = 0.5. Five independent iterations were run for each subpopulation (settings from 1 to 10) under an admixture model of population structure with correlated allele frequencies. For each iteration, a 50,000 length burn-in period and 100,000 Markov Chain Monte Carlo (MCMC) replications after burn-in were applied. STRUCTURE outputs were collated by a web-based tool STRUCTURE HARVEST (Earl, 2012) and the optimum numbers of subpopulations were chosen based on an *ad hoc* Δk statistic method (Evanno et al., 2005). The output of STRUCTURE HARVEST was imported into the program Clumpp v1.1.2 (Jakobsson and Rosenberg, 2007), which generates the population structure matrix (*Q*) based on multiple independent replicates of STRUCTURE runs. *Q* bar graphs were depicted by the program Distruct v1.1 (Rosenberg, 2004). To examine the genetic divergence among subpopulations, fixation index (*F_ST_*) was measured among each pair of subpopulations by JMP^®^ Genomics v6.0.

The pairwise kinship matrix (*K*) among all the 182 accessions was estimated using the identity-by-state (IBS) setting incorporated in JMP^®^ Genomics v6.0. After setting *r^2^*= 1.0 of tagger function implemented in HAPLOWVIEW v4.2, a subset of 3,022 unique tag-SNPs were chosen to calculate the *K-*matrix, which was then incorporated in the mixed linear model (MLM) marker-trait association (MTA) tests. To confirm the population stratification results made by Bayesian model-based clustering, hierarchical cluster analysis was conducted in JMP^®^ Genomics 6.0 using the Fast Ward algorithm method.

### Linkage Disequilibrium (LD)

JMP^®^ Genomics v6.0 was used to calculate squared Pearson’s correlation coefficient (*r^2^*) of LD among pair-wise SNP markers and fit the LD decay curve by a smoothing spline regression line (λ = 10,000) at the genome level. The pair-wise LD was evaluated for all of the 7,867 polymorphic SNP markers. The population-specific critical value of *r^2^*, which is an evidence of genetic linkage, was determined by taking the 95th percentile of *r^2^* distribution of unlinked SNP pairs following the method described in Breseghello and Sorrells (2006). The intersection of the fitted curve with this *r^2^* threshold was considered as the average estimated LD extent in the genome. Also, the mean *r^2^* and the percentage of significant marker pairs (*P* < 0.01) for each chromosome and genome were calculated to compare the degree of LD among chromosomes and genomes.

The intersection of LD decay curve and the standard critical *r^2^* = 0.30 (Maccaferri et al., 2015b; Bulli et al., 2016) was estimated at 2.25 cM. Therefore, the window size of quantitative trait loci (QTL) determined in the Ethiopian durum wheat panel was a genetic distance of ±2.25 cM from the peak of the significant associations.

### Genome-Wide Association Analyses

A total of 7,867 high-quality SNP markers and 182 accessions were used in this association analysis. R package GAPIT (Lipka et al., 2012) software was applied to analyze the associations between SNP markers and stripe rust reaction data. Strong population structure in this panel was revealed by the model-based Bayesian clustering and the IBS-based hierarchical clustering. To reduce the spurious associations caused by population structure, MLM with *Q* and *K* as covariates (Yu et al., 2005) was applied for all GWAS analyses. Loci with marker-wise significance of *P* < 0.005 were reported for the seedling resistance loci. To determine the confidence interval for the potential QTL identified in this study, multiple SNPs significantly associated to the disease response that were co-locating together and/or adjacent within intervals inferior to the general LD-decay distance of ±2.25 cM from the association peak were considered as tagging a single QTL region. For each QTL region, the SNP with the *P*-value showing the strongest association was considered as the QTL-representative marker (tag-SNP).

To identify race-specific seedling resistance loci, GWAS was performed on each race response data set separately. To detect field resistance loci, two steps of GWAS were conducted for each environment and BLUPs data of IT and SEV across all environments. The first GWAS used all 182 accessions and the second GWAS used 127 accessions whose IT of BLUPs were greater than three. The purpose of the second GWAS was to detect loci of small effects that may be hidden by the larger effect loci of seedling or field resistance. Loci that were significant (*P* < 0.005) in at least two environments and BLUPs data were reported for the field resistance. Since plant height and heading date may interact with stripe rust reaction at the adult-plant stage, GWAS was also conducted on these traits. Common significant loci (*P* < 0.005) with field resistance and these two traits were eliminated from the reports of field resistance.

### Alignments of Identified Significant Loci to Known Stripe Rust Resistance Genes and QTL

An integrated map of hexaploid wheat, consisting of published stripe rust resistance genes and QTL as well as various types of molecular markers (Rosewarne et al., 2013), was generated by Maccaferri et al. (2015b). Since the map positions of the resistance QTL identified in this durum wheat panel were derived from a tetraploid wheat consensus map (Maccaferri et al., 2015a), the first step used was to align the map positions of the identified QTL in the integrated map to those in the consensus tetraploid map, and a second step was conducted to compare the positions of the identified QTL with those of previously published *Yr* genes and QTL in the integrated map (Maccaferri et al., 2015b; Bulli et al., 2016). The alignment results are presented in Supplementary Table S6.

## Results

### Phenotypic Variation for Stripe Rust Resistance

**FIGURE 1:**
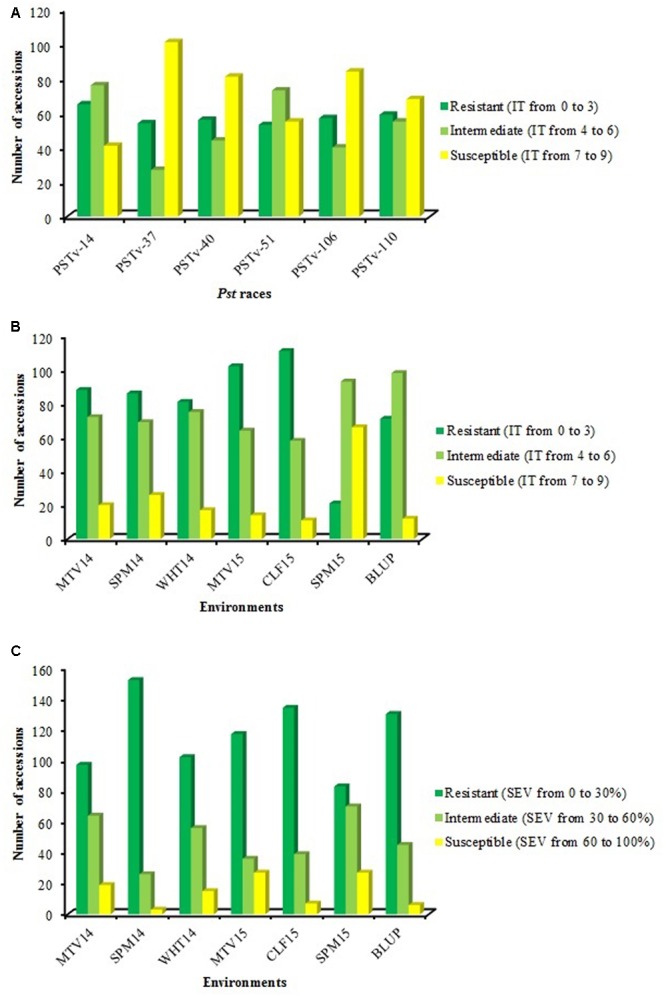
**Reaction distributions of 182 Ethiopian durum wheat landraces to *Puccinia striiformis* f. sp. *tritici*: (A)** infection type (IT) to PSTv-14, PSTv-37, PSTv-40, PSTv-51, PSTv-106, and PSTv-110, **(B)** IT and **(C)** disease severity (SEV) in individual environments as well as best linear unbiased predictor (BLUP) across all environments at the adult stages.

Single *Pst* race evaluation during the seedling stage was conducted using four races that were commonly detected in the United States in recent years and two races that predominated in Ethiopia in 2013–2014. The distributions of reaction to the six *Pst* races are summarized in **Figure 1A** and ITs of 182 accessions to individual races are presented in Supplemental Data Sheet 1. No evident differences were observed in the frequency of resistance and susceptibility to races collected from the United States and Ethiopia. About 36, 30, 31, and 29% of accessions were resistant (IT = 0 to 3) to US races PSTv-14, PSTv-37, PSTv-40 and PSTv-51, and the similar percentages of accessions were resistant to PSTv-106 and PSTv-110 from Ethiopia, with 31 and 32%, respectively. Higher percentages of accessions were susceptible (IT = 7–9) to US races PSTv-37 (56%), PSTv-40 (45%) and Ethiopian race PSTv-106 (46%) than to the US races PSTv-14 (23%), PSTv-51 (30%) and Ethiopian race PSTv-110 (37%). Among the 182 accessions, six were resistant and four were susceptible to all six races tested.

Highly significant differences were observed among accessions and accessions by environments (*P* < 0.0001) in ANOVA analyses for stripe rust response in the field tests, but no significant difference was detected among environments. Heritabilities of IT and SEV across six environments were high, at 0.87 for IT and 0.86 for SEV. Significant (*P* < 0.0001) correlations of IT and SEV were detected in the six environments, with correlation coefficient values ranging from 0.42 to 0.88, with an average of 0.59 (**Table 2**).

**Table 2:** Pearson’s correlation coefficients for infection type (IT) and disease severity (SEV) to *Puccinia striiformis* f. sp. *tritici* at the adult-plant stages in six environments.

IT vs. IT	MTV14-IT	SPM14-IT	WHT14-IT	MTV15-IT	CLF15-IT	SPM15-IT
MTV14_IT		0.71	0.60	0.47	0.54	0.49
SPM14_IT			0.71	0.55	0.61	0.64
WHT14_IT				0.44	0.52	0.58
MTV15_IT					0.61	0.47
CLF15_IT						0.51
SPM15_IT						

**SEV vs. SEV**	**MTV14-SEV**	**SPM14-SEV**	**WHT14-SEV**	**MTV15-SEV**	**CLF15-SEV**	**SPM15-SEV**

MTV14_SEV		0.59	0.56	0.53	0.52	0.54
SPM14_SEV			0.71	0.61	0.73	0.66
WHT14_SEV				0.54	0.66	0.71
MTV15_SEV					0.58	0.53
CLF15_SEV						0.68
SPM15_SEV						

**SEV vs. IT**	**MTV14-IT**	**SPM14-IT**	**WHT14-IT**	**MTV15-IT**	**CLF15-IT**	**SPM15-IT**

MTV14_SEV	0.82	0.65	0.55	0.45	0.44	0.44
SPM14_SEV		0.81	0.68	0.55	0.61	0.50
WHT14_SEV			0.88	0.45	0.53	0.49
MTV15_SEV				0.84	0.60	0.42
CLF15_SEV					0.82	0.48
SPM15_SEV						0.72

*The *P*-values of all the Pearson’s correlation coefficients are smaller than 0.0001.*

Infection type (**Figure 1B**) and SEV (**Figure 1C**) of this durum population displayed a broad and continuous distribution in the field. Relatively high frequencies of resistant accessions were observed in MTV14, SPM14, WHT14, MTV15 and CLF15, with 48, 47, 45, 56, and 61% of accessions classified as resistant, respectively. Across the six environments, 29, 61, and 10% of accessions showed resistant, intermediate resistant, and susceptible responses, respectively. The mean IT of this panel across six environments ranged from 3.3 to 5.5 and mean SEV ranged from 14.6 to 32.5%. Considering all phenotypic data, three accessions, ‘WC-2no.100,’ ‘WC-4no.78’ and ‘2000/01population37-30BDIno.63,’ were resistant at both seedling and adult-plant stages.

### Population Structure

**FIGURE 2:**
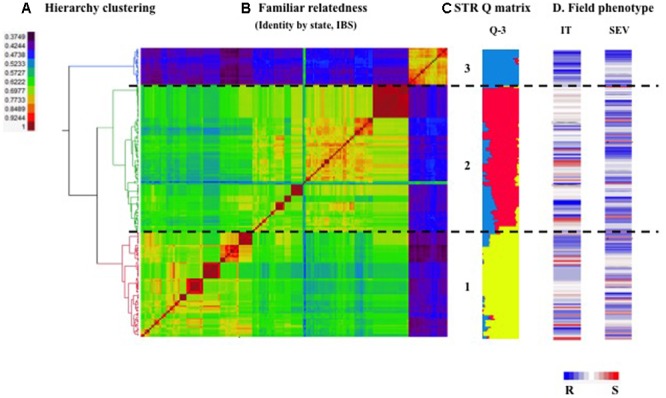
**Population structure of 182 Ethiopian durum wheat accessions. (A)** Hierarchy cluster analysis of 182 Ethiopian durum landraces using the method of identity-by-state (IBS) algorithm. **(B)** Heat map of kinship matrix using the IBS method. Horizontal dashed line divided the 182 accessions into three subpopulations based on the hierarchy clustering on the left. **(C)** Bar plot of the population structure. Accessions in **(A,B)** were arranged according to the order of kinship heat map. The colored lines represent the membership of each accession in the STRUCTURE-inferred subpopulations. **(D)** Heat map of stripe rust responses [best linear unbiased predictors for infection type (IT) and disease severity (SEV) across six environments] in the field. Blue to white to red lines show resistance to intermediate to susceptibility to stripe rust of the corresponding accessions.

The optimal number of subgroups in the durum wheat panel was determined to be three based on the Δk method of model-based Bayesian clustering analysis using STRUCTURE (Evanno et al., 2005). Subpopulation one, two and three contained 71, 86, and 25 accessions, respectively. A similar clustering result was obtained using the distance-based Fast Ward clustering method (**Figure 2**) with a high correlation (95.8%) between these two approaches. Among 22 cultivars included in this panel, 21 were grouped into subpopulation three, which accounts for 84% of the subpopulation three accessions. One cultivar was grouped into subpopulation one. Landraces comprised 100% of accessions in subpopulation two.

The groups of geographic origins admixed into the marker-based clusters. Accessions in subpopulation one were sampled from Bale (59%), DZARC (15%), Wello (11%), Gojam (6%), Tigray (4%), Shoa (3%), and Bichena (1%). Landrace accessions in subpopulation two comprised accessions from Bale (28%), Wello (28%), Akaki (23%), USA (13%), Bichena (7%), and DZARC (1%). In subpopulation three, cultivars released by DZARC and SARC comprised 56 and 28% of accessions, and landraces collected from Bale, Akaki, and Gojam comprised 8, 4, and 4% of accessions, respectively. It was noteworthy that some sampling regions were aggregated into specific marker-based clusters. All accessions originating from the Shoa and Tigray regions were grouped into subpopulation one. The accessions originating from the USA and SARC were all grouped into subpopulation two and three, respectively.

The maker-based familial relatedness was estimated by IBS and a heat map of the kinship matrix is presented in **Figure 2B**. Observing the heat map, subpopulation one and two had a relatively high level of genetic similarity compared to the other pair-wise comparison subgroups. This result was confirmed by a lower *F_ST_* value between subpopulation one and two (Supplementary Table S3).

### Linkage Disequilibrium

Linkage disequilibrium *r^2^* values among SNP marker pairs were estimated for all chromosomes. For the whole durum wheat genome, 54% of the pair-wise LD comparisons were significant at *P* < 0.01, with the mean *r^2^* of 0.116. A genome had 56% significant marker pairs and an average *r^2^* of 0.122, and B genome had 54% significant marker pairs and an average *r^2^* of 0.108. Chromosomes 1A (63%) and 5A (61%) had the highest percentage of significant marker pairs of LD *r^2^*, whereas chromosomes 6B (50%) and 7A (46%) showed the lowest.

**FIGURE 3:**
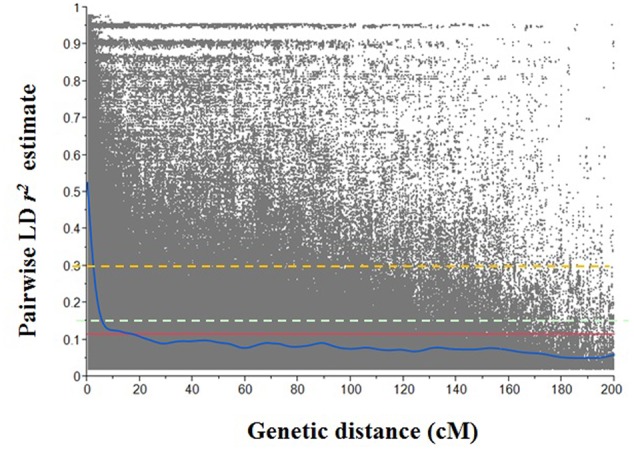
**Scatter plot of pairwise single nucleotide polymorphism (SNP) linkage disequilibrium (LD) *r^2^* over inter-marker genetic distance of the Ethiopian durum wheat genome.** The blue solid curve represents the smoothing spline regression model fit to LD decay. Red solid line represents the mean *r^2^* line (*r^2^* = 0.12) of the whole genome; cyan dash line represents the 95^th^ percentile unlinked *r^2^* line (*r^2^* = 0.15) of the whole genome; orange dash line represents the standard critical *r^2^* line (*r^2^* = 0.30) of the whole genome.

In order to investigate the extent of LD in each genome, LD decay rates were evaluated at individual genome level (**Figure 3**). Considering genome-wide LD decay in this Ethiopian durum wheat population, LD decayed below *r^2^* = 0.3 at 2.25 cM. The threshold of the 95th percentile of the *r^2^* between unlinked SNP pairs was 0.15. The interaction between the LD decay curve and threshold (*r^2^* = 0.15) was 5.75 cM. The initial LD *r^2^* was 0.53 and decreased to its 50% of value at about 2.75 cM. For A genome, the LD decay curve crossed the *r^2^* = 0.15 at 5.76 cM. The LD *r^2^* decreased to its 50% of initial value (0.55) at about 3.30 cM. For B genome, the LD *r^2^* decay to 0.15 at about 6.20 cM, and LD *r^2^* declined to half of its initial value (0.49) at about 3.25 cM.

### Genome-Wide Associations

A total of 154 SNP markers distributed on all 14 chromosomes were identified to be significantly (*P* < 0.005) associated with seedling resistance for one or more *Pst* races (Supplementary Table S4) and these 154 SNP markers were grouped into 68 loci based on the confidence interval defined as the general LD-decay distance of ±2.25 cM from the association peak. Among the 68 loci, six loci showed significant associations with at least two *Pst* races (**Table 3**). Only *YrEDWL-1BS.2*, located on the short arm of chromosome 1B, was associated with both US races PSTv-14, PSTv-37 and Ethiopian race PSTv-106. The other five loci, *YrEDWL-1AS, YrEDWL-1BS.1, YrEDWL-3AS, YrEDWL-4BL* and *YrEDWL-5BL*, were exclusively associated with resistance to US races.

**Table 3:** Six loci significantly (*P* < 0.005) associated with seedling resistance against at least two races of PSTv-14, PSTv-37, PSTv-40, PSTv-51, PSTv-106, and PSTv-110 of *Puccinia striiformis* f. sp. *tritici*.

*Yr* gene			Tag-SNP^a^					-log(*P*)^h^					
Name	Chrom^b^	CI (cM)^c^	Index	Allele^d^	RAF^e^	*R^2f^*	Associated SNP^g^	PSTv-14	PSTv-37	PSTv-40	PSTv-51	PSTv-106	PSTv-110
*YrEDWL-1AS*	1AS	7.65–12.15	IWB73129	C/**T**	0.22	0.045–0.046	IWB7333	2.76	3.06	–	–	–	–
*YrEDWL-1BS.1*	1BS	3.55–8.05	IWA7398	C/**T**	0.15	0.039–0.048	NA	2.44	–	–	2.73	–	–
*YrEDWL-1BS.2*	1BS	34.85–39.35	IWB36298	A/**C**	0.18	0.086–0.117	IWB31756, IWB10480, IWB38291, IWB74352, IWB74353, IWB669	6.01	6.64	–	–	5.30	–
*YrEDWL-3AS*	3AS	35.65–40.15	IWB7165	**C**/T	0.34	0.038–0.051	NA	2.38	–	–	2.88	–	–
*YrEDWL-4BL*	4BL	108.15–112.65	IWB35335	A/**G**	0.51	0.040–0.051	NA	2.47	–	–	2.88	–	–
*YrEDWL-5BL*	5BL	202.45–206.95	IWB20223	A/**G**	0.39	0.034–0.043	NA	2.42	2.43	–	2.51	–	–

*^a^Tag-SNP is the SNP with the strongest association in the defined QTL region. ^b^Chromosome of the identified locus is based on the tetraploid wheat consensus map generated by Maccaferri et al. (2015a). ^c^Confidence interval of the identified locus derived from a genetic distance of ±2.25 cM from the tag-SNPs; confidence interval positions based on the tetraploid wheat consensus map generated by Maccaferri et al. (2015a). ^d^Resistance allele of the tag-SNP is highlighted in bold and underlined. ^e^Resistance allele frequency of the tag-SNP. ^f^Phenotypic variances explained by the identified locus calculated in MLM with *Q3* and K. ^g^Significant (*P* < 0.005) SNPs associated with the corresponding *Pst* races and fall into a genetic distance of 2.25 cM from the tag-SNPs. NA means not applicable. ^h^-log (Marker-trait association *P* values); “-” means not significant; -log (*P*) > 2.3 and > 3 correspond to *P*-values < 0.005 and < 0.001.*

Compared to the other five seedling resistance loci, *YrEDWL-1BS.2* exhibited a higher level of contribution for IT with phenotypic variances (*R^2^*) ranging from 8.6 to 11.7%. Resistance locus *YrEDWL-1BS.2* contained a block of seven SNP markers (IWB36298, IWB31756, IWB10480, IWB38291, IWB74352, IWB74353, and IWB669) in strong LD with an average LD *r^2^* of 0.98. On the short arm of chromosome 1A, *YrEDWL-1AS* showed significant associations with US races PSTv-14 and PSTv-37 and explained from 4.5 to 4.6% of the phenotypic variance. *YrEDWL-1BS.1* on the short arm of chromosome 1B, *YrEDWL-3AS* on the short arm of chromosome 3A and *YrEDWL-4BL* on the long arm of chromosome 4B were associated with US races PSTv-14 and PSTv-51, and accounted for 3.9–4.8%, 3.8–5.1%, and 4.0–5.1% of the phenotypic variances, respectively. *YrEDWL-5BL*, mapped to the distal part of the long arm of chromosome 5B. It was associated with three US races PSTv-14, PSTv-37, and PSTv-51 with *R^2^* values ranging from 3.4 to 4.3%.

Sixty-two loci were associated with response to single races at the seedling stage (Supplementary Table S4), including 13 loci with PSTv-14, 15 loci with PSTv-37, 15 loci with PSTv-40, seven loci with PSTv-51, seven loci with PSTv-106 and five loci with PSTv-110.

Two-step sequential GWAS analyses identified 24 loci that were significantly (*P* < 0.005) associated with field resistance in at least two environments as well as BLUP data across six environments at the adult stages. Six loci, *YrEDWL-1AS, YrEDWL-1BS.1, YrEDWL-1BS.*2, *YrEDWL-3AS, YrEDWL-4BL* and *YrEDWL-5BL*, also showed significant associations with IT at the seedling stage and most likely they represent valuable all-stages resistance genes. Consequently, the six loci were not considered in the pool of loci associated with putative adult plant field-resistance only. An ANOVA model with population structure (*Q*) as a covariate was used to re-test the significance of these 18 loci. Twelve loci passed the adjusted *P* < 0.05 of ANOVA test, including seven loci, *QYrEDWL-1AL, QYrEDWL-1B.1, QYrEDWL-1BL.2, QYrEDWL-2BS, QYrEDWL-4AL, QYrEDWL-5AL.1* and *QYrEDWL-5AL.2*, that were detected in the first GWAS and five loci, *QYrEDWL.par-1AS, QYrEDWL.par-2BS, QYrEDWL.par-3BL, QYrEDWL.par-4B.1* and *QYrEDWL.par-4BL.2*, that were detected in the second GWAS (**Table 4**). The *R^2^* explained by the individual loci ranged from 1.5 to 7.6% for BLUP-IT and from 1.5 to 10.2% for BLUP-SEV across all environments. The cumulative *R^2^* explained by the 12 loci were 46.3% for BLUP-IT and 46.5% for BLUP-SEV, after subtracting variances due to population structure (Supplementary Table S5).

**Table 4:** Twelve loci significantly (*P* < 0.005) associated with field resistance at the adult-plant stages in at least two environments and best linear unbiased predictions (BLUPs) data.

QTL				Tag-SNP^a^				*R^2h^* of BLUP		
Name	GWAS step^b^	Chrom^c^	CI (cM)^d^	Index	Allele^e^	RAF^f^	Associated SNP^g^	IT	SEV	Environments with significant marker-trait associations
*QYrEDWL.par-1AS*	2	1AS	13.55–18.05	IWB20818	C/**T**	0.13	NA	0.049	0.053	MTV15-IT, MTV15-SEV, SPM15-SEV, BLUPs-IT, BLUPs-SEV
*QYrEDWL-1AL*	1	1AL	109.85–114.35	IWB31208	**A**/G	0.18	NA	0.032	0.040	SPM14-SEV, WHT14-SEV, BLUPs-IT, BLUPs-SEV
*QYrEDWL-1B.1*	1	1B	56.35–60.85	IWB29292	G/**T**	0.16	IWB61058, IWB27767, IWA5278, IWA6674, IWB60913	0.036	0.032	WHT14-IT, MTV15-IT, MTV14-SEV, BLUPs-IT, BLUPs-SEV
*QYrEDWL-1BL.2*	1	1BL	103.75–108.25	IWA3341	**A**/C	0.64	IWB64037	0.037	0.035	SPM14-IT, WHT14-IT, SPM15-IT, SPM14-SEV, BLUPs-IT, BLUPs-SEV
*QYrEDWL.par-2BS*	2	2BS	3.65–8.15	IWB52168	C/**T**	0.13	NA	0.061	0.049	WHT14-IT, CLF15-IT, SPM14-SEV, SMP15-SEV, CLF15-SEV, BLUPs-IT
*QYrEDWL-2BS*	1	2BS	14.45–18.95	IWB59815	**G**/T	0.51	NA	0.026	0.042	SPM15-SEV, CLF15-SEV, BLUPs-SEV
*QYrEDWL.par-3BL*	2	3BL	185.35–189.85	IWB33031	**A**/C	0.70	NA	0.066	0.102	SPM14-IT, MTV14-SEV, SPM14-SEV, CLF15-SEV, BLUPs-IT, BLUPs-SEV
*QYrEDWL-4AL*	1	4AL	166.35–170.85	IWB2634	**C**/T	0.25	NA	0.032	0.023	SPM15-IT, WHT14-SEV, SPM15-SEV, BLUPs-IT
*QYrEDWL.par-4B.1*	2	4B	37.55–42.05	IWB47531	**A**/G	0.79	NA	0.076	0.092	MTV14-IT, SPM14-IT, SPM14-SEV, SPM15-SEV, BLUPs-IT, BLUPs-SEV
*QYrEDWL.par-4BL.2*	2	4BL	89.45–93.95	IWB74594	**C**/T	0.15	NA	0.046	0.050	MTV15-IT, MTV15-SEV, CLF15-SEV, BLUPs-IT, BLUPs-SEV
*QYrEDWL-5AL.1*	1	5AL	102.25–107.15	IWB33606	**A**/G	0.20	IWB65854, IWB75097, IWB50640	0.015	0.034	CLF15-IT, SPM15-SEV, CLF15-SEV, BLUPs-SEV
*QYrEDWL-5AL.2*	1	5AL	124.05–128.55	IWB72387	**A**/G	0.67	NA	0.023	0.038	WHT14-IT, MTV15-IT, MTV14-SEV, WHT14-SEV, SPM15-SEV, BLUPs-SEV

*^a^Tag-SNP is the SNP with the strongest association in the defined QTL region. ^b^Significant SNPs (*P* < 0.005) identified in the first step of GWAS performing on 182 accessions and the second step of GWAS performing on 127 accessions with BLUP-IT > 3. ^c^Chromosome of the identified locus is based on the tetraploid wheat consensus map generated by Maccaferri et al. (2015a). ^d^Confidence interval of *Yr* gene derived from a genetic distance of ±2.25 cM from the tag-SNPs; confidence interval based on the tetraploid wheat consensus map (Maccaferri et al., 2015a). ^e^Chromosome of the identified locus is based on the tetraploid wheat consensus map generated by Maccaferri et al. (2015a). Resistance allele of the tag-SNP is highlighted in bold and underlined. ^f^Resistance allele frequency of the tag-SNP. ^g^Significant SNPs that fall into a genetic distance of 2.25 cM from the tag-SNPs. NA means not applicable. ^h^Phenotypic variances (*R^*2*^*) of infection type (IT) and disease severity (SEV) explained by the quantitative trait locus (QTL) calculated in MLM with *Q* and *K* model.*

Profiling tightly linked or diagnostic markers for *Yr5, Yr15, Yr36*, and *Yr30/Sr2* on the Ethiopian durum wheat panel, none of these genes were detected in any of 182 accessions. In addition, MTA analyses in this study did not identify any loci that overlapped with the chromosome regions of *Yr5, Yr15, Yr36*, and *Yr30*/*Sr2.* GWAS of plant height and heading date were also conducted with the aim to check the possible impacts of these two traits on the stripe rust response at the adult stage. No common significant SNPs were detected between these two traits and field resistance.

## Discussion

Stripe rust epidemics frequently occur in Ethiopia, but to our best knowledge, information on the resistance genes in Ethiopian landraces and cultivars is limited (Hailu and Fininsa, 2007; Dawit et al., 2009, 2012; Wan et al., 2016). In the current study, a survey for sources of resistance to contemporary *Pst* races from Ethiopia (PSTv-106 and PSTv-110) and the United States (PSTv-14, PSTv-37, PSTv-40, and PSTv-51) was evaluated in a panel of 182 durum wheat landraces and cultivars sampled from major wheat-growing areas of Ethiopia. At the seedling stage, no evident difference in frequency of resistance or susceptibility to races collected from the United States and Ethiopia in this Ethiopian durum wheat population was detected, which did not agree with the previous study of Wan et al. (2016). In a bread wheat collection containing the 178 landraces and cultivars, they detected more accessions susceptible to the Ethiopian races (PSTv-106 and PSTv-41) than to the US races (PSTv-14, PSTv-37, and PSTv-40) (Wan et al., 2016). The frequency difference between two studies could be due to differences in genetic architecture of stripe rust resistance in durum and bread wheat from Ethiopia.

PSTv-51 is the most virulent race among the six races based on the virulence/avirulence formula on 18 stripe rust differentials. Unexpectedly, a relatively high number of accessions were detected to be resistant to PSTv-51 in the current durum wheat population. This finding suggests that Ethiopian durum wheat can provide effective resistance genes for PSTv-51, which is further supported by the identification of seven loci that were exclusively associated with resistance to PSTv-51 (Supplementary Table S4) and four loci that are effective against PSTv-51 and several other races (**Table 3**). A higher frequency of landraces showed resistance to all the six *Pst* races than that of cultivars at the seedling stage under greenhouse conditions.

### Population Structure and its Impact on Stripe Rust Resistance

Three distinct subgroups within this Ethiopian durum wheat association mapping population were revealed by both the model-based Bayesian clustering and the IBS-based hierarchical clustering approach. The number of genetic-based subgroups was less than the number of cultivated regions where the accessions were collected. A high level of admixture of geographic origins/cultivation regions was observed within each of the genetic-based subpopulations, indicating that an extensive exchange of germplasm between families and villages happened in Ethiopia. However, some collection regions have strong enrichment in specific genetic-based subpopulations. A similar detection was also reported in another GWAS panel of Ethiopian durum wheat by Mengistu et al. (2016), who speculated that the reasons for this enrichment might be the preferences of local farmers toward some specific wheat landraces/pedigrees, forced by local climate conditions and/or socioeconomic elements related to quality/end use.

Population structure explained 15.9 and 13.7% of phenotypic variances for IT and SEV across six environments (Supplementary Table S5), illustrating that the population structure of the current population had some associations with the stripe rust resistance in the field. The IT and SEV of subgroup three was significantly different from subgroup one and two (Supplementary Table S3). Subgroup three, comprised of 84% modern cultivars, exhibited a higher level of disease resistance to stripe rust races tested in this study than the other two subpopulations (**Figure 2D**). The high level of resistance is most likely attributed to the enrichment of favorable resistance alleles of *QYrEDWL-1B.1, QYrEDWL-2BS*, and *QYrEDWL-5AL.1* in subgroup three (**Table 5**).

**Table 5:** Favorable allele frequencies of resistance loci in the STRUCTURE-inferred subpopulations and types of germplasm.

			Subpopulation			Germplasm type	
Significant loci	Locus type	Panel^a^	One	Two	Three	Cultivar	Landrace
*QYrEDWL.par-1AS*	Field	0.14	0.03	0.08	0.68	0.68	0.07
*QYrEDWL-1AL*	Field	0.19	0.28	0.17	0.00	0.00	0.22
*QYrEDWL-1B.1*	Field	0.17	0.01	0.06	0.96	0.95	0.06
*QYrEDWL-1BL.2*	Field	0.69	0.94	0.65	0.08	0.14	0.76
*QYrEDWL.par-2BS*	Field	0.14	0.14	0.00	0.60	0.55	0.08
*QYrEDWL-2BS*	Field	0.55	0.76	0.27	0.92	1.00	0.49
*QYrEDWL.par-3BL*	Field	0.76	0.65	0.95	0.40	0.27	0.83
*QYrEDWL-4AL*	Field	0.27	0.48	0.07	0.76	0.77	0.20
*QYrEDWL.par-4B.1*	Field	0.85	0.85	0.99	0.32	0.36	0.92
*QYrEDWL.par-4BL.2*	Field	0.16	0.24	0.03	0.40	0.36	0.14
*QYrEDWL-5AL.1*	Field	0.22	0.20	0.02	0.96	0.95	0.12
*QYrEDWL-5AL.2*	Field	0.73	0.75	0.90	0.08	0.09	0.81

*YrEDWL-1AS*	Seedling	0.24	0.24	0.13	0.64	0.68	0.18
*YrEDWL-1BS.1*	Seedling	0.16	0.13	0.00	0.84	0.86	0.07
*YrEDWL-1BS.2*	Seedling	0.17	0.45	0.00	0.00	0.05	0.19
*YrEDWL-3AS*	Seedling	0.37	0.42	0.23	0.68	0.77	0.31
*YrEDWL-4BL*	Seedling	0.55	0.83	0.47	0.04	0.05	0.62
*YrEDWL-5BL*	Seedling	0.42	0.58	0.38	0.08	0.09	0.47

*^a^Resistance allele frequencies across the panel.*

### Alignment of Significant Loci with Previously Published Stripe Rust Resistance Genes and QTL

Co-linearity was found for the map positions of all the significant SNP markers in the current panel between the tetraploid wheat consensus map (Maccaferri et al., 2015a) and hexaploid integrated map (Maccaferri et al., 2015b) (Supplementary Table S6), indicating feasibility of using the integrated map for aligning significant loci of tetraploid wheat in this study. However, alignment results should be cautiously utilized in future research due to the inherent bias in the integrated and consensus maps.

Among six seedling resistance loci, *YrEDWL-1BS.2* tagged by IWB36298 was positioned into the confidence interval of *Yr64* on chromosome 1BS. Identified from Ethiopian durum wheat accession PI 331260, *Yr64* was resistant to all tested races including PSTv-14, PSTv-37, and PSTv-40 (Cheng et al., 2014). *YrEDWL-1BS.2* in this study was associated with responses to PSTv-14, PSTv-37, and PSTv-106 but not PSTv-40. The different resistance spectrum indicated that seedling resistance locus/alleles might not be the same as *Yr64* as described in Cheng et al. (2014). Four SNPs (IWB36298, IWB10480, IWB38291, and IWB669) at this locus also showed significant associations with resistance to PSTv-14 and PSTv-37 at the seedling stage in a global collection of elite durum wheat population (Liu et al., 2017), indicating that this seedling resistance locus may be widely distributed among the durum wheat germplasm. *YrEDWL-1BS.2* was significantly associated with resistance to races PSTv-14, PSTv-37 and PSTv-106, and 16% of accessions in the Ethiopian durum population carry the resistance-associated SNP marker alleles.

The objective of GWAS analyses for field resistance in this study is to identify loci consistently associated with APR. Hence, the MTAs that were consistently detected in at least two environments plus BLUP data, but not associated with seedling resistance, were further considered. A total of 12 field resistance loci were detected in the current population, and six of them co-located with previously reported APR QTL (Supplementary Table S6).

On chromosome 1B, *QYrEDWL-1B.1*, significantly associated with stripe rust resistance in three environments, was mapped within confidence intervals of *QYrdr.wgp-1B.1L* and *QYr.cim-1BS*. Both QTL were identified in hexaploid bread wheat ‘Druchamp’ and ‘Pastor,’ and are commonly present in bread and durum wheat backgrounds (Rosewarne et al., 2012; Hou et al., 2015).

Many APR QTL have been reported on the short arm of chromosome 2B (Boukhatem et al., 2002; Carter et al., 2009; Lan et al., 2010; Lowe et al., 2011; Chen et al., 2012; Mcintosh et al., 2014). Strong associations were detected in the Ethiopian durum wheat panel in the distal part of the short arm of chromosome 2B. A minor QTL for stripe rust resistance identified in the current study, *QYrEDWL.par-2BS*, resides in the chromosomal regions of *QYr.inra-2BS. QYr.inra-2BS* was detected in hexaploid bread wheat cultivar ‘Renan,’ and was stably detected for more than 4 years and expressed during earlier adult stages compared to other QTL characterized in ‘Renan’ (Dedryver et al., 2009).

On the long arm of chromosome 4A, represented by tag-SNP IWB2634, *QYrEDWL-4AL* was significantly associated with stripe rust resistance in two environments. In the same region, a recent study using the GWAS approach in a global winter wheat collection identified a stripe rust APR locus, *QYr.wsu-4A.4* tagged by IWA4651 (Bulli et al., 2016). However, IWA4651 was neither significantly associated with any phenotype in the current population, nor in LD with IWB2634, indicating that *QYrEDWL-4AL* identified in the current study is different from *QYr.wsu-4A.4*.

*QYrEDWL.par-4B.1*, spanning the centromeric region of chromosome 4B, has been mapped in the same region of previously reported *QYr-4B* in French durum wheat ‘Sachem’ (Singh et al., 2013) and *QYr.ufs-4B* in South African spring wheat ‘Palmiet’ (Agenbag et al., 2012). *QYr-4B*, tagged by wPt-0872, was a pleiotropic QTL conferring effective resistance to both stripe rust and stem rust in the field. It is difficult to clarify the relationship between *QYr-4B* and *QYrEDWL.par-4B.1* since durum wheat ‘Sachem’ was not included in the present Ethiopian durum wheat panel. Targeted comparisons of high-density mapped SNP profiles from the relevant genotypes ‘Sachem,’ ‘Palmiet’ and selected accessions of the Ethiopian tetraploid panel would help in clarifying the identity by descent relationships at the haplotype level. Additionally, in a worldwide GWAS panel of 232 elite durum wheat lines, IWB53059 was significantly associated to field resistance and fell into the mapping intervals of *QYr-4B* and *QYr.ufs-4B*, 4.8 cM away from *QYrEDWL.par-4B.1* (unpublished data). On the distal long arm region of chromosome 4B, *QYrEDWL.par-4BL.2*, tagged by IWB74594 in this study, resides in the same region where another QTL for stripe rust resistance (*QYr-4BL*) has been identified in Israeli wheat ‘Oligoculm’ (Suenaga et al., 2003). *QYr-4BL* was consistently detected to have strong associations with leaf rust and stripe rust resistance across different environments and its effect on stripe rust resistance increased as plants advanced into adult stages.

Finally, *QYrEDWL-5AL.1* identified in the current study was located within the genomic region of *QYr.cim-5AL* on chromosome 5AL. *QYr.cim-5AL* was a minor APR QTL derived from spring wheat ‘Avocet-YrA’ and effective in trials conducted in both Mexico and China (Lan et al., 2014). Whether *QYrEDWL-5AL.1* and *QYr.cim-5AL* are the same APR locus needs further investigation.

### Potentially Novel *Pst* Resistance Loci

Five loci, *YrEDWL-1AS, YrEDWL-1BS.1, YrEDWL-3AS, YrEDWL-4BL*, and *YrEDWL-5BL* on chromosome arms 1AS, 1BS, 3AS, 4BL, and 5BL were significantly associated with seedling resistance, but do not overlap with known seedling resistance genes, and therefore should be considered as newly discovered seedling resistance loci. Of particular interest, on the short arm of chromosome 3A, a stem rust seedling resistance locus, identified in an elite durum wheat panel using GWAS (Letta et al., 2014), may be linked to *YrEDWL-3AS* based on the consensus map distance of 5.0 cM between these loci. The seedling resistance to stem rust was tagged by wPt-1923 and significantly associated with seedling resistance to stem rust races TTTTF, TTKSK (Ug99) and JRCQC (Letta et al., 2014).

Six loci associated with resistance to *Pst* in field screening nurseries, *QYrEDWL.par-1AS, QYrEDWL-1AL, QYrEDWL-1BL.2, QYrEDWL-2BS, QYrEDWL.par-3BL* and *QYrEDWL-5AL.2*, are located at chromosome positions distinct from previously reported stripe rust resistance QTL. *QYrEDWL-1BL.2* co-located with SSR marker *barc81*, which has been associated with seedling resistance to stem rust races TTTTF and TTKSK (Ug99) in a GWAS population of elite durum wheat (Letta et al., 2014). Further research to validate these loci and determine if these stem and stripe rust resistance genes are tightly linked, and their linkage phase, is warranted.

Collectively, GWAS based on this Ethiopian durum wheat population resulted in the detection of five newly documented seedling resistance loci and six newly documented field resistance loci. These newly documented loci represent 83.3 and 50.0% of total identified seedling and field resistance loci characterized in this GWAS. The historical isolation of Ethiopian durum wheat germplasm from the wheat currently under cultivation in other countries (Tsegaye and Berg, 2007; Mengistu et al., 2016), combined with regular pressure from wheat rusts in this epidemic hot-spot area of East Africa (Dawit et al., 2009; Wan et al., 2016) appears to have resulted in selection for unique diversity of stripe rust resistance genes.

## Conclusion

Landraces are generally considered to harbor promising diversity for tolerance to biotic and abiotic stresses (Zeven, 1998). However, only a small portion of this genetic diversity was actually exploited in modern wheat breeding efforts to improve agronomic performance and grain yield, mainly due to the lack of comprehensive characterization of the landraces for quantitative traits including yield, disease resistance and abiotic tolerance traits (Mengistu and Pè, 2016). The present study documents that durum wheat landraces from Ethiopia exhibit a wide-range of reactions to *Pst* infection at both the seedling and adult stages. Compared with the Ethiopian durum wheat cultivars, landraces were more resistant to *Pst* races at the seedling stage, but less resistant at the adult stage. A high proportion of newly documented stripe rust resistance loci were detected in this population. This finding provides additional evidences that tetraploid wheat landraces from Ethiopia are unique sources of trait variation, particularly for rust response. Three landrace accessions, ‘WC-2no.100,’ ‘WC-4no.78’ and ‘2000/01population37-30BDIno.63,’ were highly resistant to all six races tested at seedling stage and in all six environments at adult-plant stages. They all carried the resistance alleles of *QYrEWDL-1BL.2, QYrEWDL.par-3BL, QYrEWDL.par-4B.1, QYrEWDL-5AL.2, YrEWDL-1AS*, and *YrEWDL-4BL*. These accessions might be valuable parental lines in breeding programs for introgression of stripe rust resistance.

We identified 68 seedling resistance loci, six of which were significantly associated with resistance to multiple races. *YrEDWL-1BS.2*, effective to both US and Ethiopian races, is the most promising locus that can provide a wider protection for wheat varieties against the contemporary races. Among 62 loci associated with response to single races, 12 were associated with Ethiopian races, which can be utilized by wheat breeders in Ethiopia and the neighboring countries to battle against the new races prevalent in those regions. Twelve resistance loci were consistently detected in multiple US field screening nurseries at the adult-plant stage. These loci together explained 46.3 and 46.5% phenotypic variances for IT and SEV, respectively.

One seedling and six field resistance loci overlapped with known stripe rust resistance genes and QTL. Among them, *QYrEDWL.par-4B.1* and *QYrEDWL.par-4BL.2* overlap with the pleiotropic QTL *QYr-4B* previously reported in the French durum wheat ‘Sachem’ and *QYr-4BL* in Israeli wheat ‘Oligoculm.’ Considering that *QYrEDWL.par-4B.1* has been associated with both stripe rust and stem rust resistance and *QYrEDWL.par-4BL.2* has been associated with stripe rust and leaf rust resistance in the field, this region may be a high priority pleotropic APR locus for further characterization. Resistance loci *YrEDWL-3AS* and *QYrEDWL-1BL.2*, which do not overlap with known stripe rust resistance genes, were close to or co-located with the stem rust resistance loci that detected in a GWAS population of elite durum wheat (Letta et al., 2014). Further investigation is warranted to determine if all of these loci on chromosomes 1B, 3A, and 4B represent clusters of different resistance genes or pleiotropic resistance loci.

Our future work will focus on validating the resistance loci by using bi-parental mapping populations and developing diagnostic molecular markers that can be used in MAS in wheat breeding programs in order to accelerate the introgression of the new resistance into adapted breeding wheat varieties.

## Author Contributions

MP and WL designed the study. WL and HZ conducted the phenotyping experiments. WL performed the data analysis and wrote the manuscript. WL, SR, and MM genotyped the population. MP, RT, MM, TL, and XC provided the materials. MP, RT, MM, HZ, SR, and XC edited the manuscript.

## Conflict of Interest Statement

The authors declare that the research was conducted in the absence of any commercial or financial relationships that could be construed as a potential conflict of interest.

## Footnotes

1 http://maswheat.ucdavis.edu/protocols/Sr2/
