# Supplementary material for: Novel Sources of Stripe Rust Resistance Identified by Genome-Wide Association Mapping in Ethiopian Durum Wheat (Triticum turgidum ssp. durum)
Source: Front Plant Sci. 2017 May 12;8:774. doi: 10.3389/fpls.2017.00774 (PMC5427679; doi:10.3389/fpls.2017.00774)
Supplement: Supplementary file 1 [file Table_1.DOCX]

Table S1. Predominating races of *Puccinia striiformis* f. sp. *tritici* (*Pst*) in six field screening nurseries during 2014-2015.

| ***Pst* races** | **Virulence/Avirulence formula on *Yr* genes^a^** | **Stripe rust screening nursery** |
| --- | --- | --- |
| PSTv-37 | ***6,7,8,9,17,27,43,44,Tr1,Exp2****/1,5,10,15,24,32,SP,Tye* | WHT14, SPM14, MTV14, SPM15, MTV15 |
| PSTv-41 | ***6,7,8,9,10,17,24,27,32,43,44,Tr1,Exp2****/1,5,15,SP,Tye* | MTV15 |
| PSTv-48 | ***1,6,9,Tye****/5,7,8,10,15,17,24,27,32,43,44,SP,Tr1,Exp2* | WHT14, SPM14 |
| PSTv-52 | ***6,7,8,9,17,27,43,44,Exp2****/1,5,10,15,24,32,SP,Tr1,Tye* | WHT14, SPM14, MTV14, SPM15, CLF15 |
| PSTv-71 | ***1,6,7,9,27,43,44,Exp2,Tye****/5,8,10,15,17,24,32,SP,Tr1* | MTV14 |
| PSTv-79 | ***1,7,9,44****/5,6,8,10,15,17,24,27,32,43,SP,Tr1,Exp2,Tye* | WHT14, SPM14 |
| PSTv-140 | ***1,7,9,44,Tye****/5,6,8,10,15,17,24,27,32,43,SP,Tr1,Exp2* | CLF15 |

^a^ The virulence/avirulence formula was developed according to reactions of the 18 *Yr* near isogenic lines in the ‘Avocet S’ background to *Pst* in the US: *1* = AvSYr1NIL (*Yr1*); *5* = AvSYr5NIL (*Yr5*); *6* = AvSYr6NIL (*Yr6*); *7* = AvSYr7NIL (*Yr7*); *8* = AvSYr8NIL (*Yr8*); *9* = AvSYr9NIL (*Yr9*); *10* = AvSYr10NIL (*Yr10*); *17* = AvSYr17NIL (*Yr17*), *24* = AvSYr24NIL (*Yr24*); *27* = AvSYr27NIL (*Yr27*); *32* = AvSYr32NIL (*Yr32*); *43* = AvS/IDO377s (F3-41-1) (*Yr43*); *44* = AvS/Zak (1-1-35-line1) (*Yr44*); *SP* = AvSYrSPNIL (*YrSP*); *Tr1* = AvSYrTres1NIL (*YrTr1*); *Exp2* = AvS/Exp 1/1-1 Line 74 (*YrExp2*) and *Tye* = Tyee (*YrTye*) (Wan and Chen, 2014).
