# Supplementary material for: Novel Sources of Stripe Rust Resistance Identified by Genome-Wide Association Mapping in Ethiopian Durum Wheat (Triticum turgidum ssp. durum)
Source: Front Plant Sci. 2017 May 12;8:774. doi: 10.3389/fpls.2017.00774 (PMC5427679; doi:10.3389/fpls.2017.00774)
Supplement: Supplementary file 2 [file Table_2.DOCX]

Table S2. Primer sequences of diagnostic markers for stripe rust resistance genes *Yr5*, *Yr15*, *Yr30/Sr2* and *Yr36*.

|  | **Marker** | | **Primer sequence (5'-3')** | |
| --- | --- | --- | --- | --- |
| **Gene** | **Name** | **Type** | **Forward^a^** | **Reverse** |
| *Yr5* | IWA6121 | KASP | CCAGTGCTGGTGAAAAGCGTGA[C/T] | CCAACAAAGATTGTATAGTCCGGGGTA |
|  | IWA4096 | KASP | GCCCAGCCTGTACACCC[A/G] | TTTGATCTGAGCTGTAAATGTGTCA |
| *Yr15* | barc8 | SSR | GCGGGAATCATGCATAGGAAAACAGAA | GCGGGGGCGAAACATACACATAAAAACA |
| *Yr30/Sr2* | wMAS000005 | KASP | GTGCGAGACATCCAACACTCA [C/T] | CTCAAATGGTCGAGCACAAGCTCTA |
| *Yr36* | uhw89 | SSR | TCTCCAAGAGGGGAGAGACA | TTCCTCTACCCATGAATCTAGCA |

^a^ SNPs of KASP markers are present within brackets.
