# Supplementary material for: Novel Sources of Stripe Rust Resistance Identified by Genome-Wide Association Mapping in Ethiopian Durum Wheat (Triticum turgidum ssp. durum)
Source: Front Plant Sci. 2017 May 12;8:774. doi: 10.3389/fpls.2017.00774 (PMC5427679; doi:10.3389/fpls.2017.00774)
Supplement: Supplementary file 3 [file Table_3.docx]

Table S3. Fixation index (*F_ST_*) values, probability (*P*) values of *t* tests for infection type (IT) and disease severity (SEV) between pairs of subpopulations of Ethiopian durum wheat.

| ***F_ST_*** | **Subgroup1** | **Subgroup2** | **Subgroup3** |
| --- | --- | --- | --- |
| Subgroup1 | 0.0000 | 0.2397 | 0.3873 |
| Subgroup2 |  | 0.0000 | 0.4176 |
| Subgroup3 |  |  | 0.0000 |
| ***P* values of IT** | **Subgroup1** | **Subgroup2** | **Subgroup3** |
| Subgroup1 |  | 0.0312 | 5.23E-05 |
| Subgroup2 |  |  | 1.98E-09 |
| Subgroup3 |  |  |  |
| ***P* values of SEV** | **Subgroup1** | **Subgroup2** | **Subgroup3** |
| Subgroup1 |  | 0.1583 | 1.79E-06 |
| Subgroup2 |  |  | 2.05E-12 |
| Subgroup3 |  |  |  |
