# Supplementary material for: Novel Sources of Stripe Rust Resistance Identified by Genome-Wide Association Mapping in Ethiopian Durum Wheat (Triticum turgidum ssp. durum)
Source: Front Plant Sci. 2017 May 12;8:774. doi: 10.3389/fpls.2017.00774 (PMC5427679; doi:10.3389/fpls.2017.00774)
Supplement: Supplementary file 4 [file Table_4.docx]

Table S4. Sixty-eight loci significantly (*P* < 0.005) associated with seedling resistance to races of PSTv-14, PSTv-37, PSTv-40, PSTv-51, PSTv-106 and PSTv-110 of *Puccinia striiformis* f. sp. *tritici*.

| **Tag-SNP^a^** | | | | | |  | **-log(*P*)^i^** | | | | | |
| --- | --- | --- | --- | --- | --- | --- | --- | --- | --- | --- | --- | --- |
| **Index^b^** | **Chrom^c^** | **Pos (cM)^d^** | **Allele^e^** | **RAF^f^** | ***R^2^*^g^** | **Associated SNP index^h^** | **PSTv-14** | **PSTv-37** | **PSTv-40** | **PSTv-51** | **PSTv-106** | **PSTv-110** |
| IWB73129 | 1A | 9.9 | C/**T** | 0.22 | 0.045-0.046 | IWB7333 | 2.76 | 3.06 | - | - | - | - |
| IWB28549 | 1A | 28.4 | **C**/T | 0.79 | 0.037 | IWB40979 | - | - | - | - | - | 2.36 |
| IWA4466 | 1A | 46.1 | **C**/T | 0.60 | 0.043 | IWA8035 | - | 2.95 | - | - | - | - |
| IWB6944 | 1A | 83.1 | G/**T** | 0.23 | 0.036 | NA | - | 2.55 | - | - | - | - |
| IWB27132 | 1A | 90.8 | **A**/G | 0.20 | 0.044 | IWB41872 | 2.64 | - | - | - | - | - |
| IWB47703 | 1A | 122.7 | C/**T** | 0.67 | 0.060 | IWA7316, IWB65396 | - | - | - | - | 3.92 | - |
| IWA7398 | 1B | 5.8 | C/**T** | 0.15 | 0.039-0.048 | NA | 2.44 | - | - | 2.73 | - | - |
| IWB36298 | 1B | 37.1 | A/**C** | 0.16 | 0.086-0.117 | IWB31756, IWB10480, IWB38291, IWB74352, IWB74353, IWB669 | 6.01 | 6.64 | - | - | 5.30 | - |
| IWB59152 | 1B | 84.5 | C/**T** | 0.25 | 0.045 | IWB49534, IWB71902, IWB8999, IWB27998, IWB72552 | - | 3.06 | - | - | - | - |
| IWB65956 | 1B | 106.0 | A/**G** | 0.65 | 0.050 | NA | 2.96 | - | - | - | - | - |
| IWB51279 | 1B | 115.7 | **C**/T | 0.20 | 0.040 | NA | 2.47 | - | - | - | - | - |
| IWA4031 | 1B | 122.9 | **C**/T | 0.47 | 0.037 | NA | 2.33 | - | - | - | - | - |
| IWB31715 | 1B | 136.6 | **G**/T | 0.61 | 0.050 | IWB3081 | 2.96 | - | - | - | - | - |
| IWB7897 | 2A | 107.7 | **G**/T | 0.45 | 0.035 | NA | - | 2.5 | - | - | - | - |
| IWA2231 | 2A | 162.3 | **A**/G | 0.59 | 0.032 | NA | - | 2.32 | - | - | - | - |
| IWB39075 | 2A | 194.7 | **A**/G | 0.64 | 0.046 | NA | - | - | - | 2.63 | - | - |
| IWB72157 | 2B | 12.3 | G/**T** | 0.34 | 0.044 | IWB62546 | - | - | - | - | 3.01 | - |

(continued)

| **Tag-SNP^a^** | | | | | |  | **-log(*P*)^i^** | | | | | |
| --- | --- | --- | --- | --- | --- | --- | --- | --- | --- | --- | --- | --- |
| **Index^b^** | **Chrom^c^** | **Pos (cM)^d^** | **Allele^e^** | **RAF^f^** | ***R^2^*^g^** | **Associated SNP index^h^** | **PSTv-14** | **PSTv-37** | **PSTv-40** | **PSTv-51** | **PSTv-106** | **PSTv-110** |
| IWA6768 | 2B | 24.6 | **A**/G | 0.69 | 0.031 | NA | - | - | 2.31 | - | - | - |
| IWB10669 | 2B | 78.5 | **A**/G | 0.58 | 0.032 | NA | - | - | 2.38 | - | - | - |
| IWB26859 | 2B | 84.0 | **A**/G | 0.12 | 0.038 | IWA4102 | - | - | 2.73 | - | - | - |
| IWB2460 | 2B | 103.0 | **G**/T | 0.77 | 0.045 | NA | - | - | - | 2.58 | - | - |
| IWB62622 | 2B | 114.4 | **C**/T | 0.10 | 0.038 | IWB62718, IWB62718, IWB69270 | - | - | 2.71 | - | - | - |
| IWB29531 | 2B | 151.7 | **A**/G | 0.26 | 0.048 | IWB56398 | - | 3.19 | - | - | - | - |
| IWB56627 | 2B | 166.3 | **C**/T | 0.21 | 0.053 | IWB1384, IWB14219, IWB22835, IWA1324, IWB1091 | - | - | - | - | - | 3.17 |
| IWB43464 | 2B | 169.3 | C/**T** | 0.18 | 0.040 | NA | - | - | - | 2.36 | - | - |
| IWB7165 | 3A | 37.9 | **C**/T | 0.34 | 0.038-0.051 | NA | 2.38 | - | - | 2.88 | - | - |
| IWB72476 | 3A | 159.1 | **A**/G | 0.36 | 0.039 | NA | - | - | - | 2.3 | - | - |
| IWB69546 | 3A | 165.9 | C/**T** | 0.77 | 0.043 | NA | - | - | - | - | - | 2.68 |
| IWB51785 | 3B | 3.4 | **C**/T | 0.26 | 0.033 | NA | - | 2.39 | - | - | - | - |
| IWA3260 | 3B | 16.7 | C/**T** | 0.18 | 0.042 | IWB39782 | - | 2.87 | - | - | - | - |
| IWA6780 | 3B | 28.9 | **C**/T | 0.63 | 0.043 | NA | - | - | - | - | 2.96 | - |
| IWB58033 | 3B | 108.3 | **C**/T | 0.52 | 0.040 | NA | - | - | - | - | - | 2.50 |
| IWB68921 | 4A | 10.1 | **A**/G | 0.71 | 0.034 | NA | - | 2.42 | - | - | - | - |
| IWA3993 | 4A | 18.3 | **A**/G | 0.55 | 0.046 | IWB21402 | - | - | 3.19 | - | - | - |
| IWB4316 | 4A | 38.6 | C/**T** | 0.69 | 0.036 | NA | - | - | 2.62 | - | - | - |
| IWB32882 | 4A | 41.3 | **C**/T | 0.38 | 0.040 | IWB68026 | - | - | 2.88 | - | - | - |
| IWA5490 | 4A | 47.5 | **A**/G | 0.36 | 0.032 | NA | - | - | 2.39 | - | - | - |
| IWB73506 | 4B | 0.3 | G/**T** | 0.41 | 0.044 | NA | 2.64 | - | - | - | - | - |

(continued)

| **Tag-SNP^a^** | | | | | |  |  |  | **-log(*P*)^i^** |  |  |  |
| --- | --- | --- | --- | --- | --- | --- | --- | --- | --- | --- | --- | --- |
| **Index^b^** | **Chrom^c^** | **Pos (cM)^d^** | **Allele^e^** | **RAF^f^** | ***R^2^*^g^** | **Associated SNP index^h^** | **PSTv-14** | **PSTv-37** | **PSTv-40** | **PSTv-51** | **PSTv-106** | **PSTv-110** |
| IWB72010 | 4B | 43.9 | **C**/T | 0.46 | 0.033 | IWB7594, IWB71000, IWB36244, IWB73856, IWB48418, IWB72006, IWB27969, IWB72008, IWB73050, IWB72852, IWB72007, IWB72009, IWB7160 | - | 2.71 | - | - | - | - |
| IWB72291 | 4B | 64.3 | A/**G** | 0.66 | 0.065 | IWB72630, IWB36397, IWB72629 | - | - | - | 3.52 | - | - |
| IWB71823 | 4B | 68.9 | **A**/G | 0.19 | 0.041 | IWA6808, IWA7411 | - | - | 2.94 | - | - | - |
| IWB67255 | 4B | 74.7 | C/**T** | 0.53 | 0.034 | IWB63371, IWB22172, IWA3396, IWB70856, IWB73855 | - | 3.13 | - | - | - | - |
| IWB75098 | 4B | 104.1 | **A**/G | 0.78 | 0.046 | NA | - | - | - | - | 3.11 | - |
| IWB35335 | 4B | 110.4 | A/**G** | 0.51 | 0.040-0.051 | NA | 2.47 | - | - | 2.88 | - | - |
| IWB6896 | 4B | 114.4 | **A**/G | 0.76 | 0.040 | IWB73486 | - | - | 2.88 | - | - | - |
| IWA4766 | 5A | 26.5 | **C**/T | 0.10 | 0.037 | IWB71078, IWB14353 | - | - | 2.66 | - | - | - |
| IWA3212 | 5A | 44.9 | A/**C** | 0.44 | 0.033 | NA | - | - | - | - | 2.42 | - |
| IWB46665 | 5A | 84.4 | A/**G** | 0.80 | 0.033 | IWB37620, IWB57707 | - | - | - | - | 2.43 | - |
| IWB27036 | 5B | 59.2 | A/**G** | 0.34 | 0.032 | IWB37367 | - | 2.49 | - | - | - | - |

(continued)

| **Tag-SNP^a^** | | | | | |  |  |  | **-log(*P*)^i^** |  |  |  |
| --- | --- | --- | --- | --- | --- | --- | --- | --- | --- | --- | --- | --- |
| **Index^b^** | **Chrom^c^** | **Pos (cM)^d^** | **Allele^e^** | **RAF^f^** | ***R^2^*^g^** | **Associated SNP index^h^** | **PSTv-14** | **PSTv-37** | **PSTv-40** | **PSTv-51** | **PSTv-106** | **PSTv-110** |
| IWA2133 | 5B | 77.3 | C/**T** | 0.74 | 0.056 | IWA4571, IWA4222, IWA5139 | 3.24 | - | - | - | - | - |
| IWB68333 | 5B | 117.1 | C/**T** | 0.69 | 0.041 | IWB56225 | - | - | 2.94 | - | - | - |
| IWB75132 | 5B | 159.8 | C/**T** | 0.37 | 0.035 | NA | - | 2.49 | - | - | - | - |
| IWB20223 | 5B | 204.7 | A/**G** | 0.39 | 0.034-0.043 | NA | 2.42 | 2.43 | - | 2.51 | - | - |
| IWB65129 | 6A | 6.6 | **C**/T | 0.52 | 0.034 | IWB1550, IWB25973 | - | - | 2.48 | - | - | - |
| IWA6807 | 6A | 35.3 | **C**/T | 0.71 | 0.037 | NA | 2.31 | - | - | - | - | - |
| IWA2451 | 6B | 71.9 | **C**/T | 0.26 | 0.037 | IWB3555, IWA8129, IWA3971, IWA4065, IWB42338, IWB14571, IWA434, IWA4986, IWA1545 | - | - | 2.7 | - | - | - |
| IWA4869 | 6B | 140.1 | **A**/G | 0.63 | 0.039 | NA | 2.42 | - | - | - | - | - |
| IWB71191 | 6B | 155.1 | A/**C** | 0.48 | 0.060 | IWB73072, IWB47075, IWB12584, IWB7377, IWB9479 | - | - | - | 3.28 | - | - |
| IWB12078 | 7A | 11.5 | **A**/G | 0.70 | 0.057 | NA | 3.29 | - | - | - | - | - |
| IWB24641 | 7A | 107.6 | C/**T** | 0.49 | 0.047 | IWB2862 | 2.83 | - | - | - | - | - |
| IWB25504 | 7B | 15.2 | C/**T** | 0.55 | 0.035 | IWB3243, IWB70290 | - | - | 2.58 | - | - | - |
| IWA2568 | 7B | 20.7 | **A**/G | 0.77 | 0.038 | NA | 2.38 | - | - | - | - | - |
| IWB69063 | 7B | 95.0 | A/**G** | 0.55 | 0.061 | IWB64761 | - | - | - | 3.32 | - | - |
|  |  |  |  |  |  |  |  |  |  |  |  |  |

(continued)

| **Tag-SNP^a^** | | | | | |  |  |  | **-log(*P*)^i^** |  |  |  |
| --- | --- | --- | --- | --- | --- | --- | --- | --- | --- | --- | --- | --- |
| **Index^b^** | **Chrom^c^** | **Pos (cM)^d^** | **Allele^e^** | **RAF^f^** | ***R^2^*^g^** | **Associated SNP index^h^** | **PSTv-14** | **PSTv-37** | **PSTv-40** | **PSTv-51** | **PSTv-106** | **PSTv-110** |
| IWB48253 | 7B | 111.6 | **C**/T | 0.71 | 0.033 | NA | - | 2.37 | - | - | - | - |
| IWB27833 | 7B | 120.4 | A/**C** | 0.40 | 0.039 | IWB10797 | - | - | - | - | 2.72 | - |
| IWB40237 | 7B | 129.9 | **C**/T | 0.29 | 0.032 | NA | - | 2.32 | - | - | - | - |
| IWB25131 | 7B | 175.3 | **G**/T | 0.80 | 0.038 | NA | - | - | - | - | - | 2.42 |
| IWB27252 | 7B | 186.0 | **C**/T | 0.49 | 0.046 | NA | 2.78 | - | - | - | - | - |

^a^ Tag-SNP is the SNP with the strongest association in the defined QTL region.

^b^ Indexes of SNP according to the Illumina iSelect 90K wheat assay (Wang et al*.*, 2014).

^c^ Chromosome of the identified locus is based on the tetraploid wheat consensus map generated by Maccaferri *et al*. (2015a)

^d^ Positions of the tag-SNP is based on the tetraploid wheat consensus map generated by Maccaferri et al. (2015a)

^e^ Resistance allele of the tag-SNP is highlighted in bold and underlined.

^f^ Resistance allele frequency of the tag-SNP.

^g^ Phenotypic variance explained by the identified locus calculated in MLM with Q and K as covariates.

^h^ Significant (*P* < 0.005) SNPs that fall into a genetic distance of $\pm$2.25 cM from the tag-SNP. NA means not applicable.

^i^ -log (Marker-trait association *P* values); “-” means not significant; -log (*P*) > 2.3 and > 3 correspond to *P* values < 0.005 and < 0.001.
