# Supplementary material for: Novel Sources of Stripe Rust Resistance Identified by Genome-Wide Association Mapping in Ethiopian Durum Wheat (Triticum turgidum ssp. durum)
Source: Front Plant Sci. 2017 May 12;8:774. doi: 10.3389/fpls.2017.00774 (PMC5427679; doi:10.3389/fpls.2017.00774)
Supplement: Supplementary file 5 [file Table_5.DOCX]

Table S5. Analysis of variance with population structure (*Q*) as a covariate for best linear unbiased predictors (BLUPs) of infection type (IT) and disease severity (SEV). Probability (*P*) values of population structure alone and structure plus 12 significant loci of field resistance are presented.

| **Parameter** | ***Q*** | |  | ***Q* + loci** | |
| --- | --- | --- | --- | --- | --- |
|  | **BLUPs-IT** | **BLUPs-SEV** |  | **BLUPs-IT** | **BLUPs-SEV** |
| *R^2a^* | 15.9% | 13.7% |  | 62.2% | 60.2% |
| *Q*1 | <0.0001 | <0.0001 |  | 0.3195 | 0.1247 |
| *Q*2 | <0.0001 | <0.0001 |  | 0.9877 | 0.8546 |
| IWB20818 |  |  |  | 0.0100 | 0.0375 |
| IWB31208 |  |  |  | 0.0063 | 0.0026 |
| IWB29292 |  |  |  | <0.0001 | 0.0005 |
| IWA3341 |  |  |  | 0.0076 | 0.0061 |
| IWB52168 |  |  |  | 0.0036 | 0.0588 |
| IWB59815 |  |  |  | <0.0001 | <0.0001 |
| IWB33031 |  |  |  | 0.4264 | 0.0183 |
| IWB2634 |  |  |  | <0.0001 | <0.0001 |
| IWB47531 |  |  |  | <0.0001 | <0.0001 |
| IWB74594 |  |  |  | 0.00057 | 0.0046 |
| IWB33606 |  |  |  | 0.0127 | 0.3180 |
| IWB72387 |  |  |  | <0.0001 | <0.0001 |

^a^ Total variance explained by population structure alone and structure plus 12 significant loci of field resistance.
