# Supplementary material for: Novel Sources of Stripe Rust Resistance Identified by Genome-Wide Association Mapping in Ethiopian Durum Wheat (Triticum turgidum ssp. durum)
Source: Front Plant Sci. 2017 May 12;8:774. doi: 10.3389/fpls.2017.00774 (PMC5427679; doi:10.3389/fpls.2017.00774)
Supplement: Supplementary file 6 [file Table_6.DOCX]

Table S6. Position comparison of significant resistance loci identified in the current study to previously published genes/ quantitative trait loci.

| ***Yr/*QTL name** | **Tag-SNP** | **Chrom^a^** | | **CI based on tetraploid consensus map (cM)^b^** | | **CI based on intgrated map (cM)^c^** | | **Commonly-positioned associated SNPs^d^** | | **Overlapping *Yr*/QTL^e^** | | **References^f^** | |
| --- | --- | --- | --- | --- | --- | --- | --- | --- | --- | --- | --- | --- | --- |
| *YrEDWL-1AS* | IWB73129 | 1AS | 7.65-12.15 | | 17.48-21.98 | | IWB7333 | | NA | | NA | |  |
| *QYrEDWL.par-1AS* | IWB20818 | 1AS | 13.55-18.05 | | 18.65-23.15 | | NA | | NA | | NA | |  |
| *QYrEDWL-1AL* | IWB31208 | 1AL | 109.85-114.35 | | 123.25-127.75 | | NA | | NA | | NA | |  |
| *YrEDWL-1BS.1* | IWA7398 | 1BS | 3.55-8.05 | | 36.64-41.14 | | NA | | NA | | NA | |  |
| *YrEDWL-1BS.2* | IWB36298 | 1BS | 34.85-39.35 | | 57.66-62.16 | | IWB31756, IWB10480, | | *Yr64, Yrdurum-1BS.1* | | Cheng et al., 2014; Liu et al., 2016 | |  |
|  |  |  |  |  |  |  | IWB38291, IWB74352, | |  |  |  |  |  |
|  |  |  |  |  |  |  | IWB74353, IWB669 | |  |  |  |  |  |
| *QYrEDWL-1B.1* | IWB29292 | 1B | 56.35-60.85 | | 83.04-87.54 | | IWB27767, IWA5278, | | *QYr.cim-1BS_Pastor*, *QYrdr.wgp-1B.1L* | | Rosewarne et al., 2012; Hou et al., 2015 | |  |
|  |  |  |  |  |  |  | IWA6674, IWB60913 | |  |  |  |  |  |
| *QYrEDWL-1BL.2* | IWA3341 | 1BL | 103.75-108.25 | | 116.72-121.22 | | IWB64037 | |  | |  | |  |
| *QYrEDWL.par-2BS* | IWB52168 | 2BS | 3.65-8.15 | | 32.94-37.44 | | NA | | *QYr.inra-2BS_Renan, QYrst.orr-2B.1_Stephens* | | Dedryver et al., 2009; Vazquez et al., 2012 | |  |
| *QYrEDWL-2BS* | IWB59815 | 2BS | 14.45-18.95 | | 41.80-46.30 | | NA | | NA | | NA | |  |
| *YrEDWL-3AS* | IWB7165 | 3AS | 35.65-40.15 | | 42.33-46.83 | | NA | | NA | | NA | |  |
| *QYrEDWL.par-3BL* | IWB33031 | 3BL | 185.35-189.85 | | 179.79-184.29 | | NA | | NA | | NA | |  |
| *QYrEDWL-4AL* | IWB2634 | 4AL | 166.35-170.85 | | 191.08-195.58 | | NA | | *QYr.wsu-4A.4* | | Bulli et al., 2016 | |  |
| *QYrEDWL.par-4B.1* | IWB47531 | 4B | 37.55-42.05 | | 48.59-53.09 | | NA | | *QYr-4B_Sachem, QYr.ufs-4B_Palmiet* | | Singh et al., 2013; Agenbag et al., 2012 | |  |
| *QYrEDWL.par-4BL.2* | IWB74594 | 4BL | 89.45-93.95 | | 88.13-92.63 | | NA | | *QYr-4BL_Oligoculm* | | Agenbag et al., 2012 | |  |
| *YrEDWL-4BL* | IWB35335 | 4BL | 35.65-40.15 | | 121.70-126.20 | | NA | | NA | | NA | |  |
| *QYrEDWL-5AL.1* | IWB33606 | 5AL | 89.45-93.95 | | 90.82-95.32 | | IWB65854, IWB75097, IWB50640 | | *QYr.cim-5AL* | | Lan et al., 2014 | |  |
| *QYrEDWL-5AL.2* | IWB72387 | 5AL | 124.05-128.55 | | 113.34-117.84 | | NA | | NA | | NA | |  |
| *YrEDWL-5BL* | IWB20223 | 5BL | 202.45-206.95 | | 225.06-229.56 | | NA | | NA | | NA | |  |

^a^ The same chromosome positions of the identified *Yr*/QTL based on tetraploid wheat consensus map (Maccaferri et al., 2015a) and the integrated map (Maccaferri et al., 2015b).

^b^ Confidence interval of the identified *Yr*/QTL based on the tetraploid wheat consensus map (Maccaferri et al., 2015a).

^c^ Confidence interval of the identified *Yr*/QTL based on the integrated map (Maccaferri et al., 2015b)

^d^ Associated SNP markers in the identified QTL that commonly reside in the tetraploid wheat consensus map (Maccaferri et al., 2015a) and the integrated map (Maccaferri et al., 2015b). NA means not applicable.

^e^ Previously reported stripe rust resistance that overlapped with the loci identified in the current study according to map positions in the integrated map (Maccaferri et al., 2015b). NA means not applicable.

^f^ References for the previously published stripe rust resistance genes/QTL; the orders of the references are based on the orders of the overlapping *Yr*/QTL. NA means not applicable.
